# Supplementary material for: Corneal donation for research versus for transplantation: A-year prospective study of acceptance rates in a French University Hospital
Source: PLoS One. 2020 May 21;15(5):e0233392. doi: 10.1371/journal.pone.0233392 (PMC7241724; doi:10.1371/journal.pone.0233392)
Supplement: S1 Table — (DOCX) [file pone.0233392.s001.docx]

The following additional supporting information may be found in the online version of this article:

**Table S1** **List of medical contraindications to corneal donation for transplantation in effect at the time of the study (2017)**

| **Group** | **Description** | **Comment** |
| --- | --- | --- |
| **Viral infections** | HIV, HTLV, Hepatitis B, Hepatitis C, Rabies, ongoing CMV |  |
|  | Viral meningitis |  |
|  | Progressive viral disease: herpes, chickenpox, shingles... | If positive Immunoglobulin M |
|  | History of hepatitis (according to the family interviews) | Hepatitis A excepted |
|  | Multiple sexual partners for less than 1 year |  |
|  | Intravenous or nasal drug addiction or partner of | *Except if weaning >12 months |
|  | Blood exposure accident in the last 12 months |  |
|  | Prison stay more than 4 days for less than 1 year |  |
|  | Chikungunya, West Nile, Zika, others* | *According to health alerts |
|  | Severe Influenza (“malignant flu”) | Not influenza alone |
| **Vaccines** | Recent live-attenuated vaccines (for less than one month) : rubella, measles, mumps, yellow fever, rotavirus, chickenpox, tuberculosis |  |
| **Bacterial infection** | Bacterial encephalopathy or meningitis |  |
|  | Active tuberculosis or suspicion |  |
|  | Leprosy, brucellosis, legionellosis, Lyme, leptospirosis | Except if cured |
| **Parasitic infections** | Leishmaniasis, malaria, toxoplasmosis, toxoplasmosis | Except if cured |
| **Fungal infection** | Uncontrolled fungal infection |  |
| **Risk of prion diseases** | Surgery with dura mater |  |
|  | Treatment with extractive pituitary hormone before 2000 |  |
|  | Neurosurgical intervention before 2000 |  |
|  | Neurological symptoms suggestive of Creutzfeld-Jacob disease or Bovine spongiform encephalopathy (BSE) |  |
|  | Dementia, Alzheimer's disease |  |
|  | Parkinson's disease (and any neurodegenerative disease) |  |
|  | Travel to endemic areas, including the UK between 1980 and 1996 (6 months cumulative) |  |
|  | Britishman/woman born before 1996 |  |
| **Neurological diseases** | Multiple Sclerosis, amyotrophic lateral sclerosis, neurological diseases of unknown origin |  |
|  | Parkinson's disease |  |
|  | Any neurodegenerative disease |  |
| **Cancer** | Aplasia following chemotherapy | Risk of unreliable serology |
|  | Metastatic primary brain tumor | medulloblastoma, chordoma, chordoma, glioblastoma multiforme, anaplastic oligodendroglioma, anaplastic ependynoma, anaplastic meningioma, primary lymphoma, pineoblastoma, sarcoma, grade 2 and 3 astrocytoma |
|  | Brain metastasis of unknown cancer |  |
|  | Carcinomatous meningitis |  |
|  | Melanoma and history of melanoma (w/o date limitation) |  |
|  | Myeloma, lymphoma, leukemia, Hodgkin's, myelodysplasia  (any malignant hematopathy, even cured) |  |
| **Death from unknown cause** |  |  |
| **Toxic substances** |  |  |
| **Xenograft transplant** |  |  |
| **Drugs** | Steroids > 15 days and > 1mg/Kg/d | Risk of unreliable serology |
|  | High-dose immunosuppressant or immunoglobulin | Risk of unreliable serology |
| **Risk of hemodilution** | Blood products and colloids within 48 hours prior to serological blood sampling  and/or  Crystalloids within one hour before the serological blood sampling  > 50% of plasma volume |  |
| **Age** | 0 to 2 years |  |
| **Preservation of the deceased's body** | At less than 2°C |  |
| **Clinical examination** | Traces of bites (suspicion of drug addiction) |  |
|  | Tattoo/piercing less than 3 months old |  |
|  | Local tumor |  |
|  | Lesion suggestive of melanoma |  |
|  | Local infection |  |
|  | Unexplained wound |  |
|  | Undocumented skin rash (viral risk) |  |
|  | Significant cachexia |  |
| **Local pathology** | Cataract surgery before 1994 |  |
|  | Corneal surgery including refractive laser |  |
|  | Corneal disease (Trisomy 21, Marfan...) |  |
|  | Pterygium on visual axis |  |
|  | Local irradiation |  |
|  | Lyell's Syndrome |  |
|  | History of ocular herpes |  |
