# Supplementary material for: Corneal donation for research versus for transplantation: A-year prospective study of acceptance rates in a French University Hospital
Source: PLoS One. 2020 May 21;15(5):e0233392. doi: 10.1371/journal.pone.0233392 (PMC7241724; doi:10.1371/journal.pone.0233392)
Supplement: Attachment — Submitted filename: Response to reviewers.docx [file pone.0233392.s002.docx]

PONE-D-19-35417
Corneal donation for research versus for transplantation: a-year prospective study of acceptance rates in a French University Hospital

- A rebuttal letter that responds to each point raised by the academic editor and reviewer(s). This letter should be uploaded as separate file and labeled 'Response to Reviewers'.
- A marked-up copy of your manuscript that highlights changes made to the original version. This file should be uploaded as separate file and labeled 'Revised Manuscript with Track Changes'.
- An unmarked version of your revised paper without tracked changes. This file should be uploaded as separate file and labeled 'Manuscript'.

Please note while forming your response, if your article is accepted, you may have the opportunity to make the peer review history publicly available. The record will include editor decision letters (with reviews) and your responses to reviewer comments. If eligible, we will contact you to opt in or out.

**First, we would like to thank the Academic Editor, reviewer #1 and #2 for her/his reviewing and advises. We have chosen to respond to the comments and to resubmit this manuscript. We deeply apologize for grammar or language mistakes.**

**Journal Requirements:**

When submitting your revision, we need you to address these additional requirements:

1. Please ensure that your manuscript meets PLOS ONE's style requirements, including those for file naming. The PLOS ONE style templates can be found at http://www.plosone.org/attachments/PLOSOne_formatting_sample_main_body.pdf and http://www.plosone.org/attachments/PLOSOne_formatting_sample_title_authors_affiliations.pdf

**No problem, we followed guidelines**

2. Please provide additional details regarding participant consent. In the Methods section, please ensure that you have specified (1) whether consent was informed and (2) what type you obtained (for instance, written or verbal). If your study included minors, state whether you obtained consent from parents or guardians. If the need for consent was waived by the ethics committee, please include this information.

**Consent was informed. In France we have opt out system. The type of the consent depended of each eligible deceased : written or oral refusal during lifetime for deceased, or oral consent or refusal (by phone or face to face) from relatives contacted.**

**We precised it in Methods section.**

3. Thank you for including your ethics statement: The Agence de la Biomedecine (ABM) specifically authorized corneas procurement for research (PFS15-008 & PFS16-010) and study was approved by local Institutional Review Board (IORG0007394, N°IRBN272016/CHUSTE).

Please amend your current ethics statement to include the full name of the ethics committee/institutional review board(s) that approved your specific study.

Once you have amended this/these statement(s) in the Methods section of the manuscript, please add the same text to the “Ethics Statement” field of the submission form (via “Edit Submission”).

For additional information about PLOS ONE ethical requirements for human subjects research, please refer to <http://journals.plos.org/plosone/s/submission-guidelines#loc-human-subjects-research>.

**We have amended the full name of our institutional review board that approved our specific study**  **Ethics Committee of the CHU de Saint-Etienne, Research Commission of Terre d’Ethique**

**We precised it in the Methods Section and in the “Ethics statement” field of the submission form.**

4. We note that you have a patent relating to material pertinent to this article. Please provide an amended statement of Competing Interests to declare this patent (with details including name and number), along with any other relevant declarations relating to employment, consultancy, patents, products in development or modified products etc. Please confirm that this does not alter your adherence to all PLOS ONE policies on sharing data and materials, as detailed online in our guide for authors <http://journals.plos.org/plosone/s/competing-interests> by including the following statement: "This does not alter our adherence to  PLOS ONE policies on sharing data and materials.” If there are restrictions on sharing of data and/or materials, please state these. Please note that we cannot proceed with consideration of your article until this information has been declared.

**The patent does not alter our adherence to  PLOS ONE policies on sharing data and materials.**

This information should be included in your cover letter; we will change the online submission form on your behalf. **OK no problem.**

5. Thank you for stating the following in the Competing Interests section:

"P GAIN, S ACQUART and G THURET are inventors on “patent US 20160029618A1” submitted by University Jean Monnet that covers “Medical device intended for long-term storage of a cornea, or for ex vivo experimentation on a human or animal cornea”.
P GAIN and G THURET are consultant for Thea laboratories and Quantel Medical."

We note that one or more of the authors are employed by a commercial company: Thea laboratories and Quantel Medical.

a.     Please provide an amended Funding Statement declaring this commercial affiliation, as well as a statement regarding the Role of Funders in your study. If the funding organization did not play a role in the study design, data collection and analysis, decision to publish, or preparation of the manuscript and only provided financial support in the form of authors' salaries and/or research materials, please review your statements relating to the author contributions, and ensure you have specifically and accurately indicated the role(s) that these authors had in your study. You can update author roles in the Author Contributions section of the online submission form.

Please also include the following statement within your amended Funding Statement. **OK, no problem.**

“The funder provided support in the form of salaries for authors [insert relevant initials], but did not have any additional role in the study design, data collection and analysis, decision to publish, or preparation of the manuscript. The specific roles of these authors are articulated in the ‘author contributions’ section.”

**“The funder provided support in the form of salaries for authors [PG, GT], but did not have any additional role in the study design, data collection and analysis, decision to publish, or preparation of the manuscript. The specific roles of these authors are articulated in the ‘author contributions’ section.”**

If your commercial affiliation did play a role in your study, please state and explain this role within your updated Funding Statement.

**No commercial affiliation played role in our study.**

b. Please also provide an updated Competing Interests Statement declaring this commercial affiliation along with any other relevant declarations relating to employment, consultancy, patents, products in development, or marketed products, etc.

Within your Competing Interests Statement, please confirm that this commercial affiliation does not alter your adherence to all PLOS ONE policies on sharing data and materials by including the following statement: "This does not alter our adherence to  PLOS ONE policies on sharing data and materials.” (as detailed online in our guide for authors <http://journals.plos.org/plosone/s/competing-interests>) . If this adherence statement is not accurate and  there are restrictions on sharing of data and/or materials, please state these. Please note that we cannot proceed with consideration of your article until this information has been declared.

**Commercial affiliations do not alter our adherence to  PLOS ONE policies on sharing data and materials.**

Please include both an updated Funding Statement and Competing Interests Statement in your cover letter. We will change the online submission form on your behalf.

Please know it is PLOS ONE policy for corresponding authors to declare, on behalf of all authors, all potential competing interests for the purposes of transparency. PLOS defines a competing interest as anything that interferes with, or could reasonably be perceived as interfering with, the full and objective presentation, peer review, editorial decision-making, or publication of research or non-research articles submitted to one of the journals. Competing interests can be financial or non-financial, professional, or personal. Competing interests can arise in relationship to an organization or another person. Please follow this link to our website for more details on competing interests: <http://journals.plos.org/plosone/s/competing-interests>

6. We note that you have included the phrase “data not shown” in your manuscript. Unfortunately, this does not meet our data sharing requirements. PLOS does not permit references to inaccessible data. We require that authors provide all relevant data within the paper, Supporting Information files, or in an acceptable, public repository. Please add a citation to support this phrase or upload the data that corresponds with these findings to a stable repository (such as Figshare or Dryad) and provide and URLs, DOIs, or accession numbers that may be used to access these data. Or, if the data are not a core part of the research being presented in your study, we ask that you remove the phrase that refers to these data.

**We apologize for that, we precised this point in reviewed manuscript.**

**Reviewers' comments:**

Reviewer's Responses to Questions

**Comments to the Author**

1. Is the manuscript technically sound, and do the data support the conclusions?

The manuscript must describe a technically sound piece of scientific research with data that supports the conclusions. Experiments must have been conducted rigorously, with appropriate controls, replication, and sample sizes. The conclusions must be drawn appropriately based on the data presented. 

Reviewer #1: Yes

Reviewer #2: Yes

2. Has the statistical analysis been performed appropriately and rigorously? 

Reviewer #1: Yes

Reviewer #2: Yes

3. Have the authors made all data underlying the findings in their manuscript fully available?

The [PLOS Data policy](http://www.plosone.org/static/policies.action#sharing) requires authors to make all data underlying the findings described in their manuscript fully available without restriction, with rare exception (please refer to the Data Availability Statement in the manuscript PDF file). The data should be provided as part of the manuscript or its supporting information, or deposited to a public repository. For example, in addition to summary statistics, the data points behind means, medians and variance measures should be available. If there are restrictions on publicly sharing data—e.g. participant privacy or use of data from a third party—those must be specified.

Reviewer #1: Yes

Reviewer #2: Yes

4. Is the manuscript presented in an intelligible fashion and written in standard English?

PLOS ONE does not copyedit accepted manuscripts, so the language in submitted articles must be clear, correct, and unambiguous. Any typographical or grammatical errors should be corrected at revision, so please note any specific errors here.

Reviewer #1: Yes

Reviewer #2: Yes

5. Review Comments to the Author

Please use the space provided to explain your answers to the questions above. You may also include additional comments for the author, including concerns about dual publication, research ethics, or publication ethics. (Please upload your review as an attachment if it exceeds 20,000 characters)

Reviewer #1: The study is technically sound with a valid scientific question. The results were interesting and surprising. We note that the paper did not mention whether the research cornea donors were tested for relevant communicable diseases and what the disposition of the corneas would be should the results come back positive. Was there any concern that some of the cognitive disorders were due to prion disease?

**Thank you for your comment, we have added the following sentences in the discussion section (lines 291 to 307)**

**“only rabies (constantly fatal), herpes simplex virus, bacteria, fungi and retinoblastoma have been formally demonstrated to be transmissible via the cornea. So, targeting these contraindications allows researchers to benefit from them. Some sporadic cases of prion disease were reported in recipients, and circumstantial evidence has implicated corneal transplantation as a mechanism of transmission of iatrogenic prion disease : transplantation is presumed (but not formal) to be the source of prion disease in recipients [31-34]. Although we could be concern about disease transmission or research bias induced by these contraindications, it clearly depends on the research aim(s). In this study, every cornea was procured by in situ excision with single-use sterile instrument, without any contact with retina or optic nerve, which are be considered as specific risk factors to possible iatrogenic spread of sporadic and variant prion disease [35, 36].”**

Our other query would be the consent taking process for biomedical research, perhaps this can be mentioned in the paper? Will this be different from the consent for clinical transplant purposes? Or can we assume that ABM has already provided the necessary authorization and therefore no further consent is required?

**Thank you for your comment. We clarified this point lines 122 to 139.**

The manuscript is easily understood but there is room for improvement with regard to overall sentence construction, grammatical flow and word choices. **We deeply apologize for grammar or language mistakes. We updated this point.**

Agree with the author’s view that the high consent rates may be a single centre effect and may not represent the situation in most other institutions around the world. However, it echoes the need for a consistent and sustained messaging to the general public about cornea donation, whether it be for therapy, research or education.

Reviewer #2: In this prospective study, the authors compared the donation acceptance rate for cornea transplantation (deceased patients with no medical contraindication for corneal transplantation) and for research (deceased patients with contraindication for corneal transplantation) and evaluated if the acceptance rate for transplantation donation remained stable over a year.

General comments

Obtaining fresh corneas for research is highly challenging and a main concern for worldwide researchers. While it is highly interesting to see that acceptance rates for research and for transplant were comparable, this paper opens several questions regarding research regulations of corneas. Indeed, in France, historically, ineligible corneas for transplant were being used for research, in consequence, research corneas came from patients without medical contraindication for transplant.

In the present study, corneas from deceased patients with medical contraindication for transplant (including cognitive disorders, blood cancers, neurologic disorder, uncontrolled infection, tumor of CNS, severe influenza and hemodilution) were used for research to increase the number of fresh corneas needed for research purpose.
The main question here might be “should we use tissues from cognitive disorders, blood cancers, neurologic disorder, tumor of CNS, uncontrolled infections… for our research?” Indeed, the authors explained that one of the main focus of their lab was to develop an active storage machine for corneal graft. We could be concern about both disease transmission and induced research bias which is not at all discussed in the paper. **Thanks for this comment. We added new comment concerning this point. (cf. response to comment of reviewer #1 about prion disease => line 291 to 307)**

Thus, the discussion should address those points which can’t be only resumed in one sentence (Line 279-281) “The medical contraindications to corneal donation for transplantation are numerous, based mainly on precautionary principle solely and deprive recipients of potentially great intrinsic quality corneas”. **We added new comment concerning this point (line 291 to 307).**

Was any risk analysis performed before the use of those corneas? **Yes, We added new comment concerning this point (line 303 to 307).**

**“We did risk analysis before the use of those corneas to develop our active storage machine to make evolve eyebanking, with assessment of their baseline intrinsic quality (endothelial cell density, transparency, presence of scar or not, presence of previous refractive surgery or not) and safety (no infection), following tests used in daily routine in eyebanks.”**

Point by point comments

Some additional points need to be clarified and are listed below.

Results

Line 153 the authors reported 1442 intra-hospital deaths but only 371 deceased eligible. Can the authors explain the reasons of the gap since cornea for research are based on medical contraindications meaning that families of all deceased patients should be interviewed. What were the limitations? **, We added new comment concerning this point (line 371 to 380).**

**Gap can be explained by several causes :**

**1/ legislative framework with death-to-procurement time >24h, which discards deceased from eligibility to for corneal donation for research or transplantation (n= 413)**

**2/ fast transport of the deceased body from hospital mortuary to private funeral homes or conservative care, which prevent from interviewing families for research or transplantation (n=411)**

**3/ Only 3 nurses of the coordination team are present to do all the work about corneas but also for other tissues (vessels, bone) and organs (mainly kidney). So, sometimes they do not have enough time to treat every dossier of eligible deceased for research or for transplantation in their daily tasks (between corneas, other tissues, organs) (n=243) ; so** **increase the resources allocated to the coordinators is crucial.**

**4/ medico-legal impediment with prosecutor objection (n=4).**

Table 1 is nonreadable, please correct. In addition, in the table, the total of research interviews is 126 but number written in results line 154 is 127. Please review and correct. **We apologize for that ; we reviewed and corrected on revised manuscript.**

Line 212 “medical contraindications that allowed selection in the research group” can you please clarify the French rules about research on tissues with medical contraindications for transplant?

**The Rules are edited and updated regularly by the Health Authorities, and this is The Agence de la biomédecine provides the rules : for each tissue medical contraindications exist for transplantation. Ineligibility for transplantation makes tissue discarded and so destructed. But some of this discarded tissue which does not represent infectious risks, may be used by specific, certified and authorized laboratories. For ocular tissue, distribution of tissue are free of charge in France.**

End of comments

6. PLOS authors have the option to publish the peer review history of their article ([what does this mean?](https://journals.plos.org/plosone/s/editorial-and-peer-review-process#loc-peer-review-history)). If published, this will include your full peer review and any attached files.


If you choose “no”, your identity will remain anonymous but your review may still be made public.


**Do you want your identity to be public for this peer review?** For information about this choice, including consent withdrawal, please see our [Privacy Policy](https://www.plos.org/privacy-policy).

Reviewer #1: Yes: Howard Cajucom -Uy

Reviewer #2: No

[NOTE: If reviewer comments were submitted as an attachment file, they will be attached to this email and accessible via the submission site. Please log into your account, locate the manuscript record, and check for the action link "View Attachments". If this link does not appear, there are no attachment files to be viewed.]

While revising your submission, please upload your figure files to the Preflight Analysis and Conversion Engine (PACE) digital diagnostic tool, <https://pacev2.apexcovantage.com/>. PACE helps ensure that figures meet PLOS requirements. To use PACE, you must first register as a user. Registration is free. Then, login and navigate to the UPLOAD tab, where you will find detailed instructions on how to use the tool. If you encounter any issues or have any questions when using PACE, please email us at [figures@plos.org](mailto:figures@plos.org). Please note that Supporting Information files do not need this step.
