# Supplementary material for: Corneal donation for research versus for transplantation: A-year prospective study of acceptance rates in a French University Hospital
Source: PLoS One. 2020 May 21;15(5):e0233392. doi: 10.1371/journal.pone.0233392 (PMC7241724; doi:10.1371/journal.pone.0233392)
Supplement: Attachment — Submitted filename: R2_Response to reviewers.docx [file pone.0233392.s003.docx]

PONE-D-19-35417R1
Corneal donation for research versus for transplantation: a-year prospective study of acceptance rates in a French University Hospital
PLOS ONE

Saint-Etienne, May 1st 2020

Dear Professor Yu-Chi Liu,

Please find attached a revised version of our article titled “Corneal donation for research versus for transplantation: a-year prospective study of acceptance rates in a French University Hospital”.

We have carefully considered and responded to all the points addressed by the reviewers.

Per your instructions, all substantive amendments in the revised version are stated in our point-by-point response, and are marked in red in the article.

We greatly hope that this new version will meet the reviewers' expectations and comply with your editorial policy.

Yours sincerely,

Dr. Thibaud GARCIN, M.D., Ph.D., FEBO

Saint-Etienne University Hospital

"Corneal Graft Biology, Engineering, and Imaging" Laboratory EA 2521

Faculty of Medicine

Saint-Etienne

France

Dear Dr. GARCIN,

Thank you for submitting your manuscript to PLOS ONE. After careful consideration, we feel that the manuscript has significantly improved but the reviewers still have some minor concerns. Therefore, we invite you to submit a revised version of the manuscript that addresses the points raised during the review process.


We would appreciate receiving your revised manuscript by Jun 14 2020 11:59PM. When you are ready to submit your revision, log on to <https://www.editorialmanager.com/pone/> and select the 'Submissions Needing Revision' folder to locate your manuscript file.

If you would like to make changes to your financial disclosure, please include your updated statement in your cover letter.

To enhance the reproducibility of your results, we recommend that if applicable you deposit your laboratory protocols in [protocols.io](http://protocols.io), where a protocol can be assigned its own identifier (DOI) such that it can be cited independently in the future. For instructions see: <http://journals.plos.org/plosone/s/submission-guidelines#loc-laboratory-protocols>

Please include the following items when submitting your revised manuscript:

- A rebuttal letter that responds to each point raised by the academic editor and reviewer(s). This letter should be uploaded as separate file and labeled 'Response to Reviewers'.
- A marked-up copy of your manuscript that highlights changes made to the original version. This file should be uploaded as separate file and labeled 'Revised Manuscript with Track Changes'.
- An unmarked version of your revised paper without tracked changes. This file should be uploaded as separate file and labeled 'Manuscript'.

Please note while forming your response, if your article is accepted, you may have the opportunity to make the peer review history publicly available. The record will include editor decision letters (with reviews) and your responses to reviewer comments. If eligible, we will contact you to opt in or out.

We look forward to receiving your revised manuscript.

Kind regards,

Yu-Chi Liu, M.D
Academic Editor
PLOS ONE

Reviewers' comments:

Reviewer's Responses to Questions

**Comments to the Author**

1. If the authors have adequately addressed your comments raised in a previous round of review and you feel that this manuscript is now acceptable for publication, you may indicate that here to bypass the “Comments to the Author” section, enter your conflict of interest statement in the “Confidential to Editor” section, and submit your "Accept" recommendation.

Reviewer #1: All comments have been addressed

Reviewer #2: (No Response)

2. Is the manuscript technically sound, and do the data support the conclusions?

The manuscript must describe a technically sound piece of scientific research with data that supports the conclusions. Experiments must have been conducted rigorously, with appropriate controls, replication, and sample sizes. The conclusions must be drawn appropriately based on the data presented. 

Reviewer #1: Yes

Reviewer #2: Partly

3. Has the statistical analysis been performed appropriately and rigorously? 

Reviewer #1: Yes

Reviewer #2: Yes

4. Have the authors made all data underlying the findings in their manuscript fully available?

The [PLOS Data policy](http://www.plosone.org/static/policies.action#sharing) requires authors to make all data underlying the findings described in their manuscript fully available without restriction, with rare exception (please refer to the Data Availability Statement in the manuscript PDF file). The data should be provided as part of the manuscript or its supporting information, or deposited to a public repository. For example, in addition to summary statistics, the data points behind means, medians and variance measures should be available. If there are restrictions on publicly sharing data—e.g. participant privacy or use of data from a third party—those must be specified.

Reviewer #1: Yes

Reviewer #2: Yes

5. Is the manuscript presented in an intelligible fashion and written in standard English?

PLOS ONE does not copyedit accepted manuscripts, so the language in submitted articles must be clear, correct, and unambiguous. Any typographical or grammatical errors should be corrected at revision, so please note any specific errors here.

Reviewer #1: Yes

Reviewer #2: Yes

6. Review Comments to the Author

Please use the space provided to explain your answers to the questions above. You may also include additional comments for the author, including concerns about dual publication, research ethics, or publication ethics. (Please upload your review as an attachment if it exceeds 20,000 characters)

Reviewer #1: (No Response)

Reviewer #2: Discussion
Line 268 misspelling of “iin” please replace by “in”
This has been corrected

The authors may want to add some additional information regarding the CoVid current situation which will probably change their future practice. In their study, all deceased patients were eligible for research donation, will they now consider testing all deceased patients before harvesting corneas? Are French rules currently changing regarding cornea donation for research?

Thank you for the suggestion. The continuation of corneal procurement as early as possible is indeed crucial both for patients waiting for transplants and for laboratories like ours that work on these irreplaceable human tissues. The SARS-Cov-2 epidemic has effectively stopped all but multi-organ donation. It appears that we may soon be able to resume therapeutic procurement from symptomatic, non-at-risk individuals, but there are many unanswered questions.

In order to be able to answer the question of the risk of donor-recipient transmission, **we have already started a new research work and obtained the authorization from our health authority (Biomedicine Agency, PFS-2020-011) to resume retrieval for scientific purposes for the next 150 donors.** Thus, we will systematically collect and test all donors (nasopharynx, conjunctiva and cornea) and carry out serologies. This will provide us with robust data to make good decisions about what new tests to perform or not to perform on donors, for transplantation and for research. We hope that the new recommendations will be based on evidence and not on an unfounded precautionary principle.

Of course, during this new study we are recording the acceptance rate for corneal donation among all potential donors, and possible reasons for refusal. We will know whether the current epidemic is changing behavior and we will submit a letter to report the results, if you wish.

For this article, as suggested, we would like to add the following paragraph as the end of discussion:

“By the time this article is accepted, the global SARS-Cov-2 epidemic has stopped corneal procurement for therapeutic and scientific purposes altogether. Scientific knowledge is sorely lacking to establish the risk of transmission of this virus via ocular tissues and therefore to make new recommendations on which virological tests should be added or not to ensure total safety. Our French health authority has authorized us in emergency (PFS-2020-011) to perform a large series of scientific procurements from any potential donor (COVID+ or -) in order to objectively analyze the risks. During this particular study we will also analyze whether the epidemic has modified the acceptance rate and the reasons for a possible refusal.”

Respectfully

Dr T Garcin, MD, PhD., FEBO

7. PLOS authors have the option to publish the peer review history of their article ([what does this mean?](https://journals.plos.org/plosone/s/editorial-and-peer-review-process#loc-peer-review-history)). If published, this will include your full peer review and any attached files.


If you choose “no”, your identity will remain anonymous but your review may still be made public.


**Do you want your identity to be public for this peer review?** For information about this choice, including consent withdrawal, please see our [Privacy Policy](https://www.plos.org/privacy-policy).

Reviewer #1: Yes: Howard Cajucom-Uy

Reviewer #2: No


[NOTE: If reviewer comments were submitted as an attachment file, they will be attached to this email and accessible via the submission site. Please log into your account, locate the manuscript record, and check for the action link "View Attachments". If this link does not appear, there are no attachment files to be viewed.]
